# Pituitary insufficiency with masked inflammation: Pituitary abscess

**DOI:** 10.1002/ccr3.5943

**Published:** 2022-06-02

**Authors:** Ryohei Ono, Sho Nishiguchi, Izumi Kitagawa

**Affiliations:** ^1^ Department of General Internal Medicine Shonan Kamakura General Hospital Kamakura City Japan

**Keywords:** fever, inflammatory marker, panhypopituitarism, pituitary abscess

## Abstract

A 29‐year‐old woman presented with fever and amenorrhea. Laboratory findings showed no elevation inflammatory markers; however, hormonal evaluation revealed panhypopituitarism. She was finally diagnosed with pituitary abscess, and underwent transsphenoidal excision. The patient was treated with antibiotics and oral hormonal supplementation, and her pituitary function finally normalized.

## Graphical abstract

The symptoms such as fever and leukocytosis are seen in only one third of patients with pituitary abscess. Physicians should take care of pituitary insufficiency even without obvious fever or elevation of inflammatory markers.

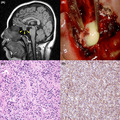

## 1 INTRODUCTION

**FIGURE 1:**
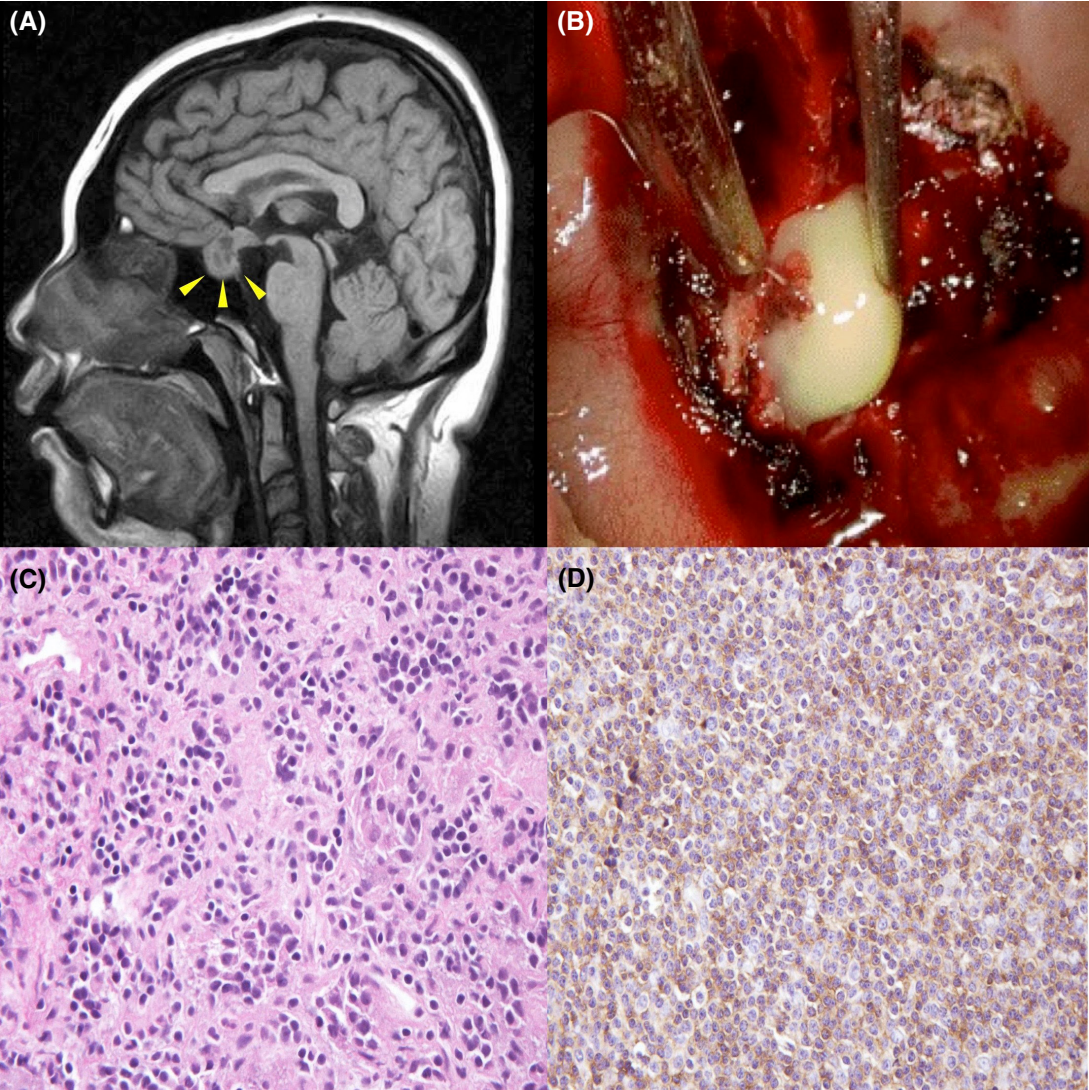
(A) Pituitary enhanced T1‐weighted magnetic resonance imaging revealing a cystic mass with a high intensity signal in the pituitary fossa (arrows). (B) Operative findings showing yellowish pus of the pituitary gland. Pathological findings of the resected pituitary gland showing abscess formation with neutrophil and lymphocyte infiltration (C; Hematoxylin and eosin stain, D; Immunostaining for leukocyte common antigen)

A 29‐year‐old woman presented with a three‐month history of amenorrhea and a two‐month history of mild fever. She was treated with acetaminophen; however, the symptoms continued. Physical examinations were unremarkable except for bitemporal side of blurred vision. Laboratory findings showed no elevation of white blood cells (6000/μl) and C‐reactive proteins (0.02 mg/dl); however, hormonal evaluation revealed panhypopituitarism (Table 1). No pathogen was isolated from the blood. Pituitary enhanced T1‐weighted magnetic resonance imaging (MRI) revealed a cystic mass with hypo‐intense in the pituitary fossa (Figure 1A). She underwent transsphenoidal excision, and the pituitary gland was filled with yellowish pus (Figure 1B). Pathological findings showed abscess formation with numerous neutrophils and lymphocytes infiltration (Figure 1C,D).

**TABLE 1:** Laboratory findings

	Value with units	Normal range
Prolactin	158 ng/ml	6.1–30.5
Luteinizing hormone	1.5 IU/L	1.8–10.2
Follicle‐stimulating hormone	3.9 IU/L	3.0–14.7
Thyroid‐stimulating hormone	0.02 μIU/ml	0.38–4.31
Free thyroxine	0.42 ng/dl	0.82–1.63
Morning serum cortisol	0.6 μg/dl	4.5–21.1
Adrenocorticotropic hormone	8.8 pg/dl	7.2–63.3

### 1.1 Question

What is the diagnosis?

### 1.2 Answer

Pituitary abscess.

She was finally diagnosed with pituitary abscess (PA). The patient was treated with intravenous antibiotics and oral hormonal supplementation of glucocorticoids, levothyroxine, and desmopressin, and her pituitary function finally normalized after two weeks.

Pituitary abscess is a very rare disease accounting for 0.2%–0.6% of all pituitary lesions and usually presents with unspecific symptoms. The symptoms such as fever and leukocytosis are seen in only one third of the PA patients.^1^ Typical MRI features of PA include a cystic mass that appears hypo‐intense in T1‐weighted. Physicians should take into consideration in differential diagnosis of PA in patient with pituitary insufficiency even without obvious fever or elevation of inflammatory markers.^2^

## AUTHOR CONTRIBUTIONS

RO contributed to conception and design of case report; acquisition, analysis, and interpretation of data; and drafting and revising the article. SN and IK contributed to patient management, analysis and interpretation of data, and revising the article. All authors gave final approval of the article and have agreed to be accountable for all aspects of the work.

## CONFLICT OF INTEREST

None.

## ETHICAL APPROVAL

Not applicable.

## CONSENT

Written informed consent was obtained from the patient to publish this report in accordance with the journal's patient consent policy.

## Data Availability

Not applicable.
